# Levetiracetam mitigates doxorubicin-induced DNA and synaptic damage in neurons

**DOI:** 10.1038/srep25705

**Published:** 2016-05-11

**Authors:** Jose Felix Moruno Manchon, Yuri Dabaghian, Ndidi-Ese Uzor, Shelli R. Kesler, Jeffrey S. Wefel, Andrey S. Tsvetkov

**Affiliations:** 1 Department of Neurobiology and Anatomy, University of Texas, Houston Medical School, Houston, TX, USA; 2 The Jan and Dan Duncan Neurological Research Institute, Baylor College of Medicine, Houston, TX, USA; 3 Department of Computational and Applied Mathematics, Rice University, Houston, TX, USA; 4 The University of Texas Graduate School of Biomedical Sciences, Houston, TX, USA; 5 Department of Neuro-Oncology, M.D. Anderson Cancer Center, Houston, TX, USA

## Abstract

Neurotoxicity may occur in cancer patients and survivors during or after chemotherapy. Cognitive deficits associated with neurotoxicity can be subtle or disabling and frequently include disturbances in memory, attention, executive function and processing speed. Searching for pathways altered by anti-cancer treatments in cultured primary neurons, we discovered that doxorubicin, a commonly used anti-neoplastic drug, significantly decreased neuronal survival. The drug promoted the formation of DNA double-strand breaks in primary neurons and reduced synaptic and neurite density. Pretreatment of neurons with levetiracetam, an FDA-approved anti-epileptic drug, enhanced survival of chemotherapy drug-treated neurons, reduced doxorubicin-induced formation of DNA double-strand breaks, and mitigated synaptic and neurite loss. Thus, levetiracetam might be part of a valuable new approach for mitigating synaptic damage and, perhaps, for treating cognitive disturbances in cancer patients and survivors.

Patients may suffer cognitive changes during and after anti-cancer treatments^1^,^2^. Up to 75% of people who undergo chemotherapy experience some level of cognitive changes^2^,^3^. Chemotherapy often affects patients’ attention, memory, and processing speed. Those changes may go away soon after chemotherapy is over or can persist for years^3^. Several factors are believed to contribute to cancer-related cognitive dysfunction, including direct effects of cancer^4^, age^5^, genetic risk factors^6^, immune responses^1^,^7^, and—most importantly—the direct effects of anti-neoplastic drugs^1^,^3^,^8^.

Doxorubicin (Dox) is a commonly used anti-neoplastic agent for treating breast and other cancers^9^. Decline in cognitive function has been observed in more than 60% of breast cancer patients treated with Dox^9^. In the nucleus, Dox intercalates into DNA, leading to the eviction of the histone proteins from chromatin^10^. Dox also inhibits the enzyme topoisomerase II, which relaxes supercoils in DNA for transcription^11^. In cultured neurons, Dox affects long-term enhanced excitability, long-term synaptic facilitation, and long-term synaptic depression^12^. Dox has a poor penetration into the brain, but still, it appears to penetrate the brain at levels sufficient to cause neurotoxicity and to damage neural stem cells^13^,^14^.

DNA damage and repair occur in post-mitotic neurons under physiologic brain activity^15^. Aging and age-associated disorders enhance neuronal DNA damage^16^,^17^,^18^. Neurons treated with amyloid-beta, a peptide critically involved in the pathogenesis of Alzheimer’s disease (AD), exhibit more DNA double-strand breaks (DSBs), based on the accumulation of phosphorylated histone H2A variant X (γH2A.X)^15^, and less BRCA1, a protein that repairs DSBs^19^. Abnormally increased numbers of DSBs and downregulated BRCA1 are thought to be associated with synaptic dysfunction^15^,^19^. Remarkably, levetiracetam, an anti-epileptic drug, normalizes levels of γH2A.X in amyloid-beta-treated neurons and in a mouse model of AD, the hAPP mouse line^15^. Furthermore, the drug reverses synaptic and cognitive deficits in the hAPP mice, suggesting that levetiracetam might be a therapy for AD^15^,^20^,^21^. Lamotrigine, another anti-epileptic drug, also prevents the loss of dendritic spines and attenuates the deficits in learning and memory in mouse models of AD^22^.

In this study, we determined if Dox induces the DSBs in primary cultured neurons and if levetiracetam reduces formation of these DNA breaks and mitigates neuronal damage. We first demonstrated that Dox significantly decreased neuronal survival. We also discovered that Dox promoted accumulation of γH2A.X in the nuclei, reflecting enhanced DNA damage, and downregulated BRCA1. Remarkably, Dox accumulated in the nuclei of cultured neurons. The drug also damaged neurites and synapses in cultured neurons. Pre-treatment with levetiracetam, an FDA-approved anti-epileptic drug, mitigated Dox-induced DNA damage. The drug also alleviated the synaptic and neurite count lost to Dox treatment. Based on our findings, we conclude that levetiracetam might assist in the development of therapies for chemotherapy-induced cognitive impairment.

## Results

### Dox Reduces Neuronal Survival

**Figure 1:**
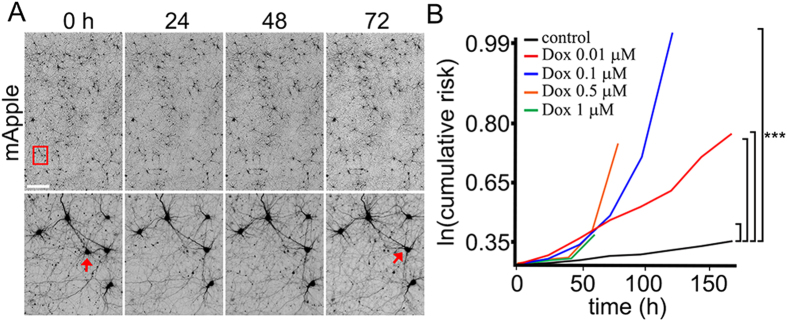
Dox reduces neuronal survival. (**A**) An example of survival analysis. Primary cortical neurons transfected with mApple were tracked with an automated microscope. Images collected after 24 hours demonstrate the ability to return to the same field of neurons and follow them over time. Each image is a montage of non-overlapping images captured in one well of a 24- or 96-well plate. Scale bar is 300 μm. (**B**) Risk of death associated with Dox treatment of primary cortical neurons. Four cohorts of neurons expressing mApple were treated with 0.01, 0.1, 0.5, and 1 μM Dox. Treatment with Dox led to increased cell death. All neurons in three cohorts (0.1, 0.5, and 1 μM Dox) died before the end of the experiments. ***p < 0.001 (Log-Rank test).

Cognitive impairments in patients treated with Dox suggest that the drug affects neuronal homeostasis^23^. To determine if Dox is toxic for cultured neurons in our system, we treated primary cortical neurons with a vehicle or with different doses of Dox and measured the cumulative risk of neuronal death with an automated microscopy and longitudinal analysis^24^,^25^. This technique enables us to monitor large cohorts of individual neurons over their lifetimes and to measure their survival with the statistical approaches used in clinical medicine. By tracking neurons over their lifetimes, we can determine whether applied drugs contribute positively or negatively or neutrally to neuronal fate. Neurons were transfected with the mApple construct to visualize morphology. Dox or vehicle was added, and the mApple-expressing neurons were tracked for 7 days (Fig. 1A). Loss of the red fluorescence is a sensitive marker of neuronal death^24^,^26^. By analyzing when each neuron lost its fluorescence, we can measure neuronal survival with cumulative hazard statistics (Fig. 1A). We found that treatment with Dox enhanced neuronal death at nanomolar concentrations (Fig. 1B). These data are in an agreement with our previous findings that Dox induces significant cognitive impairments and affects brain network connections^27^.

### Dox Induces DNA DSBs and Downregulates BRCA1 in Primary Neurons

**Figure 2:**
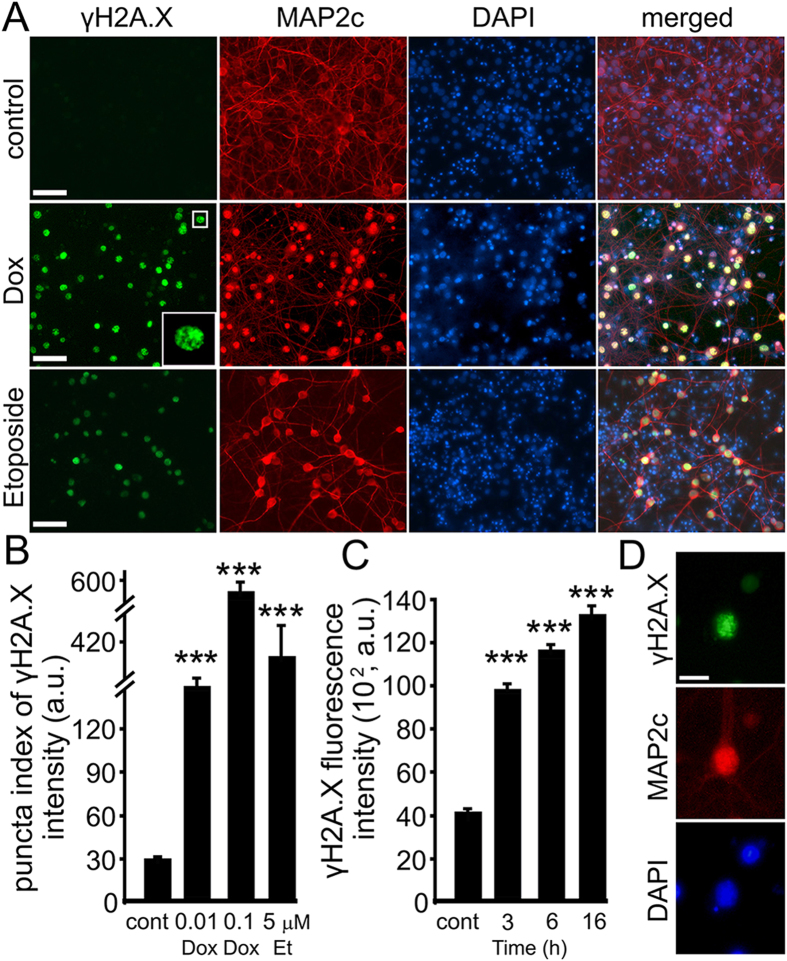
Dox promotes formation of DNA DSBs in primary neurons. (**A**) Cortical neurons at 28–32 DIV were treated with a vehicle or with Dox (0.1 μM) or with DNA damaging drug etoposide (5 μM) overnight, fixed, and stained for a marker of DSBs phosphorylated histone H2A variant X, γH2A.X (green), MAP2c (red), and with the nuclear Hoechst dye (blue), and imaged. The neuronal nucleus is enlarged on the Dox panel to illustrate the γH2A.X puncta. Note the green nuclear staining in cells treated with Dox and etoposide. Also note the reduced dendritic arborization in neurons treated with Dox and etoposide. Scale bar is 20 μm. (**B**) Images of fixed neurons treated with Dox (0.1 μm) overnight, Dox (0.01 μM) for 3 days, or etoposide (5 μM) overnight were analysed with an automated algorithm. The puncta index was estimated by measuring the standard deviation of γH2A.X fluorescence intensity. The puncta index of γH2A.X staining is increased in neurons treated with Dox and etoposide. ***p < 0.0001 (Dunnett’s test). A.u., arbitrary units. Several thousand neurons were analyzed. Results were pooled from at least three independent experiments. (**C**) Images of fixed neurons treated with Dox (0.1 μm) for indicated times were analysed for DNA DSBs. The levels of γH2A.X were estimated by measuring the γH2A.X fluorescence intensity. ***p < 0.0001 (Dunnett’s test). (**D**) An example of a neuron from cultures treated with Dox (0.1 μm) for 6 hours, and stained as in (**A**). Note γH2A.X puncta in the nucleus. Scale bar is 5 μm.

Dox induces DNA damage in cancer cells^28^. We, therefore, tested if treatment of cultured primary neurons with Dox results in accumulation of a marker of DSBs, phosphorylated histone H2A variant X (γH2A.X). To determine if Dox damages neuronal DNA, synaptically developed neurons were treated with Dox. In parallel, some neurons were independently treated with etoposide, a drug commonly used for inducing DNA damage in cells^29^. Neurons were fixed and stained for γH2A.X and MAP2c. Neurons treated with either Dox or etoposide had enhanced staining of γH2A.X in neuronal nuclei, and non-treated cultures rarely had γH2A.X-positive neurons (Fig. 2A,B). Remarkably, Dox was the most damaging compound. DNA damage was evident after 3 hours of incubation with Dox (Fig. 2C,D).

**Figure 3:**
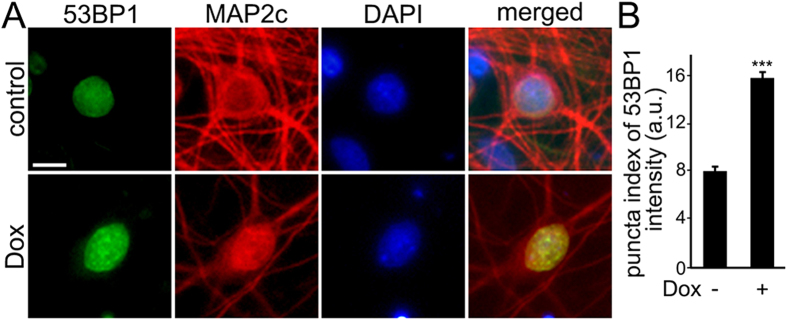
Dox induces formation of 53BP1-positive puncta. (**A**) Cortical neurons at 28–32 DIV were treated with a vehicle or with Dox (0.1 μM) overnight, fixed, and stained for a marker of DNA damage 53BP1 (green), MAP2c (red), and with the nuclear Hoechst dye (blue), and imaged. (**B**) The puncta index was estimated by measuring the standard deviation of 53BP1 fluorescence intensity. The puncta index of 53BP1 staining is increased in neurons treated with Dox. ***p < 0.0001 (t-test). A.u., arbitrary units. One hundred and fifty neurons were analyzed. Results were pooled from two independent experiments.

A critical regulator of the cellular response to DNA DSBs is p53-binding protein 1 (53BP1), which acts as a scaffold that recruits additional proteins to damaged DNA. We determined if treatment of primary neurons with Dox results in the formation of 53BP1-positive puncta, which reflect the formation of DNA DSBs, as in non-neuronal cells^30^. Neurons were fixed and stained for 53BP1 and MAP2c. Neurons treated with Dox had 53BP1 localized to puncta. In control neurons, 53BP1 was diffuse, as expected (Fig. 3A,B). Based on these data, we conclude that Dox induces the accumulation of DNA DSBs in post-mitotic neurons.

**Figure 4:**
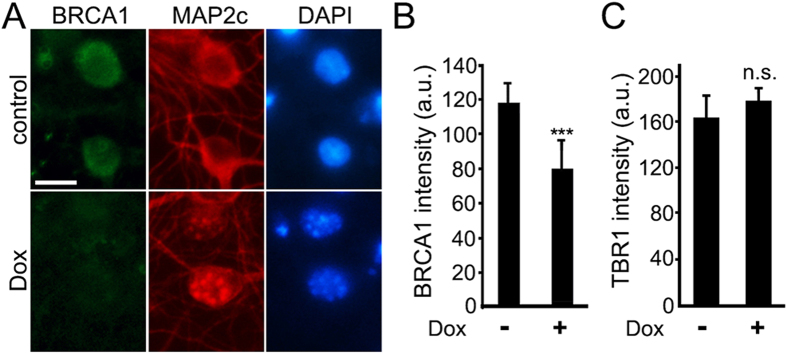
Dox downregulates BRCA1 in primary neurons. (**A**) Cortical neurons at 28–32 DIV were treated with a vehicle or with Dox (0.1 μM) for 2 days, fixed, and stained for BRCA1 (green), MAP2c (red), and the nuclear Hoechst dye (blue), and imaged. Note that, although BRCA1 is mostly nuclear, there is some perinuclear BRCA1 staining, which was observed in neurons before^19^. Scale bar is 5 μm. (**B**) Images of fixed neurons treated with Dox (0.1 μm) from (**A**) were analysed. ***p < 0.0001 (t test). (**C**) Cortical neurons were treated with a vehicle or with Dox (0.1 μM) for 2 days, fixed, and stained for TBR1 (green), MAP2c (red), and with the nuclear Hoechst dye (blue), and imaged. Images were analysed for the nuclear TBR1 fluorescence intensities. N.S., non-significant. A.u., arbitrary units. One hundred neurons were analysed. Results were pooled from two independent experiments.

A recent paper demonstrated that BRCA1, a protein that repairs DNA DSBs in non-neuronal cells, also repairs DNA DSBs in neurons. Interestingly, amyloid-beta and neuronal activity downregulate BRCA1 leading to the formation of DNA DSBs. Knocking down BRCA1 in neurons causes DNA DSBs, neuronal shrinkage, synaptic impairments, and learning and memory deficits, but not apoptosis^19^. BRCA1 is also downregulated in brains of patients with AD^15^,^19^. We therefore hypothesized that Dox may also have an effect on BRCA1 in neurons. Synaptically developed neurons were treated with Dox or with a vehicle. Neurons were fixed and stained for BRCA1 and MAP2c. Neurons treated with Dox had less staining of BRCA1, than non-treated neurons (Fig. 4A,B). The observed effect was not a general downregulation of the nuclear proteins, because steady-state levels of TBR1, a nuclear protein that regulates neuronal identity^31^, were not affected by Dox (Fig. 4C). Based on this finding, we conclude that Dox downregulates BRCA1 in neurons, which perhaps leads to the accumulation of DNA DSBs.

### Dox Accumulates in the Nuclei of Neurons

While analyzing fixed neurons with a fluorescent microscope, we noticed an unusual red MAP2c staining of neurons treated with Dox (Figs 2A,D, 3A and 4A). This red nuclear staining was absent in neurons treated with etoposide or with a vehicle. MAP2c is a microtubule-associated protein, which localized to neuronal processes, and is excluded from the nucleus. It is unlikely that Dox causes the relocalization of MAP2c to the nucleus.

**Figure 5:**
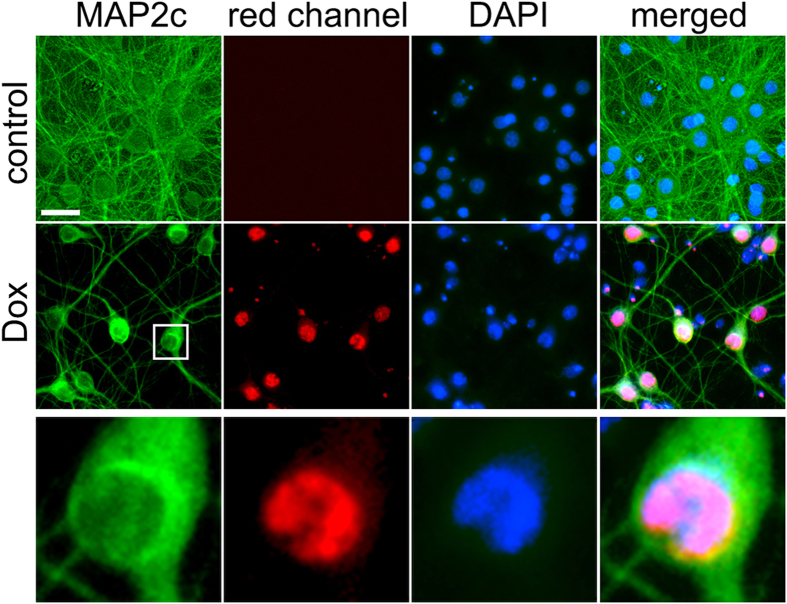
Dox accumulates in the neuronal nuclei. Cortical neurons at 28–32 DIV were treated with Dox (0.01 μM) for two days, fixed, and stained for MAP2c (Alexa Fluor 488; green) and with the nuclear Hoechst dye (blue), and imaged. Note the red nuclear staining in cells treated with Dox. Scale bar is 20 μm. The zoomed image demonstrates the red nuclear staining.

Dox has two nicknames “red evil” or “red death”, perhaps, due to the side effects that it causes and due to its red color^32^,^33^. Dox’s spectrum has an emission peak at 595 nm and a shoulder at 650 nm^34^. Therefore, we hypothesized that the nuclear red “MAP2c” staining that we observed might be due to Dox’s red fluorescence. To confirm this, we treated neurons with a vehicle or with Dox for 2 days, fixed, and stained with an antibody for MAP2c, and with the DAPI dye. The secondary antibodies against the MAP2c antibodies were Alexa Fluor 488-labeled (green). As expected, we did not observe any red signal in vehicle-treated neurons. Interestingly, we did observe bright red nuclear signal in Dox-treated neurons (Fig. 5). We conclude that Dox indeed accumulates in the nucleus, where it perhaps physically interacts with DNA by intercalation.

### Reducing Neuronal Activity Mitigates the Formation of DNA DSBs

**Figure 6:**
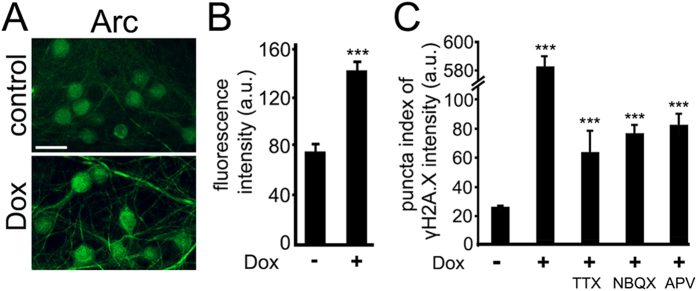
Reducing neuronal activity mitigates the formation of DNA DSBs mediated by Dox. (**A**) Dox upregulates Arc in neurons. Cortical neurons at 28–32 DIV were treated with a vehicle or with Dox (0.1 μM) overnight, fixed, and stained for Arc (green), and imaged. Scale bar is 25 μm. (**B**) Images of fixed neurons from (**A**) were analysed. ***p < 0.0001 (t test). One hundred neurons were analysed. Results were pooled from three independent experiments. (**C**) Cortical neurons at 28–32 DIV were treated with a vehicle or with Dox (0.1 μM) or with Dox (0.1 μM) and TTX (1 μM), or with Dox (0.1 μM) and NBQX (50 μM), or with Dox (0.1 μM) and with APV (100 μM) overnight, fixed, and stained for γH2A.X, MAP2c, and with the nuclear Hoechst dye, imaged, and analyzed with an automated algorithm. The puncta index was estimated by measuring the standard deviation of γH2A.X fluorescence intensity. *** p < 0.00001 (t-test). A.u., arbitrary units. Several thousand neurons were analyzed. Results were pooled from at least three independent experiments.

Some level of DNA DSBs and their repair occur in neurons under physiologic neuronal activity^15^. Toxic agents, such as amyloid-beta, aging and age-associated disorders, increase neuronal DNA damage, which is attributed to aberrant neuronal activity. Suppressing the aberrant activity prevents the abnormal formation of DNA DSBs. First, we tested if Dox indeed modulates neuronal activity. Arc, a protein required for memory formation and synaptic plasticity, is upregulated in response to prolonged increased activity^35^,^36^,^37^,^38^,^39^. Synaptically developed neurons were treated with Dox or with a vehicle. Neurons were fixed and stained for Arc. Neurons treated with Dox had increased staining of Arc, suggesting that Dox stimulates neuronal activity (Fig. 6A,B).

Second, to determine if inhibiting neuronal activity prevents Dox-induced DNA DSBs, we co-treated neurons with Dox and a sodium channel blocker tetrodotoxin (TTX) or with Dox and an antagonist of AMPA receptors NBQX or with Dox and an antagonist of NMDA receptors APV (Fig. 6C). TTX reduced the formation of DNA DSBs induced by Dox, suggesting that the effect of Dox partially depends on neuronal activity and release of neurotransmitters (Fig. 6C). NBQX reduced the formation of DNA DSBs induced by Dox, indicating that AMPA receptor–mediated postsynaptic depolarization might also be involved (Fig. 6C). APV mitigated the formation of DNA DSBs induced by Dox, suggesting the involvement of NMDA receptors, at least partially (Fig. 6C). We, therefore, conclude that Dox partially induces the formation of DNA DSBs by enhancing neuronal activity.

### Dox Affects Synaptic and Neurite Density in Cultured Neurons

**Figure 7:**
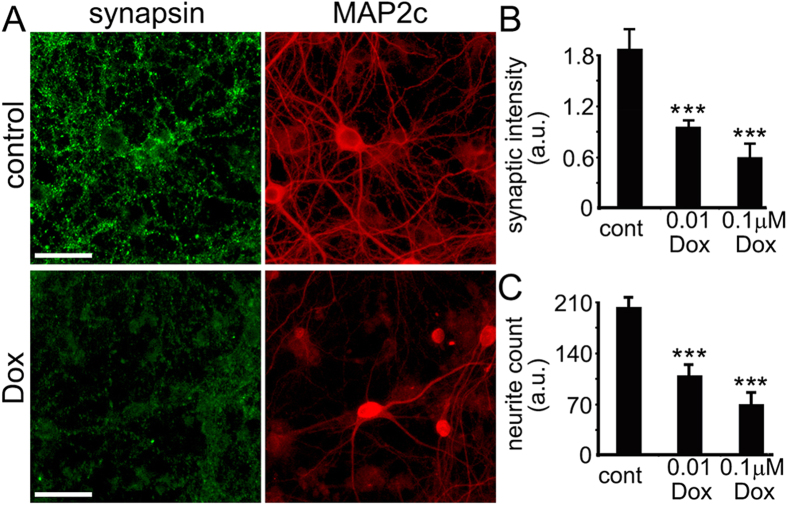
Dox damages synapses and neurites in cultured primary cortical neurons. (**A**) Synaptically developed primary cortical cultures at 28–32 DIV were treated with a vehicle overnight (upper panel) or with 0.01 μM Dox for two days (lower panel), fixed, and stained with antibodies against synapsin and MAP2c. Scale bar is 20 μm. (**B**) Quantification of fluorescent images of cortical cultures treated with a vehicle or with Dox (0.01 μM for 3 days, or 0.1 μM, overnight), and stained with antibodies against synapsin (green) and microtubule-associated protein 2, MAP2c, (red). Synapsin staining was used by the algorithm to identify and analyze synapses. ***p < 0.0001 (Dunnett’s test). Cont., control. A.u., arbitrary units. Several thousand neurons were analyzed. Results were pooled from at least three independent experiments. Note that red MAP2c staining also includes Dox’s red fluorescence. (**C**) Quantification of fluorescent images from (**B**) with an algorithm that identifies and analyzes neurites. MAP2c staining was used by the algorithm to identify and analyze neurites (cont., control). ***p < 0.0001 (Dunnett’s test). Cont., control. A.u., arbitrary units.

Cognitive abilities depend on effective synaptic function. Commonly used anti-neoplastic agents or their metabolites directly interact with synaptic components, such as synaptic receptors and enzymes^40^,^41^,^42^,^43^,^44^,^45^,^46^. In addition, chemotherapy drugs may alter synapses by reducing the health of mitochondria at the synapse^47^,^48^. Theoretically, anti-neoplastic agents can affect the transcription of synaptic genes. We hypothesized that Dox affects synapses in cultured primary neurons. To test this hypothesis, we cultured rat embryonic cortical neurons for 4 weeks to allow them to synaptically develop, treated them with Dox, fixed the neurons, stained with antibodies against synapsin and a neuronal marker MAP2c, and analyzed them with fluorescent microscopy. We found that Dox significantly reduced the density of synapses and neurites (Fig. 7A–C). Therefore, together with DNA damage, this likely contributes to neuronal dysfunction and cognitive impairments in cancer patients and survivors.

### Levetiracetam as a Potential Neuroprotectant

**Figure 8:**
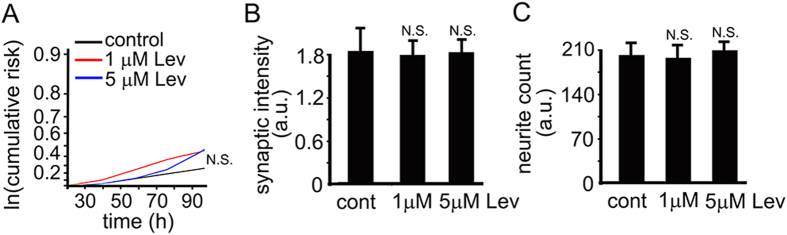
Levetiracetam as a potential neuroprotectant. (**A**) Risk of death associated with levetiracetam (Lev) treatment of primary cortical neurons. 1–5 μM had no effect on baseline risk of death. N.S., not significant. Results were pooled from at least three independent experiments. (**B**) Quantification of fluorescent images of cortical cultures at 28–32 DIV treated with a vehicle or with 1–5 μM levetiracetam (Lev) for 3 days, and stained with antibodies against synapsin and microtubule-associated protein, MAP2c. Synapsin staining was used by the algorithm to identify and analyze synapses. Cont., control. Levetiracetam, Lev. A.u., arbitrary units. N.S., not significant (t-test). (**C**) Quantification of fluorescent images from (**C**) with an algorithm that identifies and analyzes neurites based on MAP2c staining. Cont., control. Levetiracetam, Lev. A.u., arbitrary units. N.S., not significant (t-test).

Antiepileptic drugs, such as levetiracetam or lamotrigine, which prevent aberrant neuronal activity, are neuroprotective in cell and animal models of neurodegeneration and neuronal injury^20^,^21^,^22^,^49^. Importantly, levetiracetam reduces the DNA damage caused by amyloid-beta and reverses synaptic and cognitive deficits in an AD mouse model^15^,^20^,^21^. We hypothesized that levetiracetam would prevent or mitigate DNA damage and synaptic abnormalities induced by Dox in our neuronal model. Before testing that, we decided to determine a safe dose of levetiracetam in our cultures that could potentially protect neurons without causing overt toxicity itself. To determine if levetiracetam is toxic for cultured neurons, we treated primary cortical neurons with 1–5 μM levetiracetam or vehicle and measured the cumulative risk of neuronal death with automated microscopy and longitudinal analysis^24^. Neurons were transfected with mApple, drug or vehicle was added, and the mApple-expressing neurons were tracked for several days (Fig. 8A). As expected, levetiracetam was well tolerated (Fig. 8A). We also tested if levetiracetam affects synaptic and neurite density. Levetiracetam did not change synapses and neurites in cultured neurons (Fig. 8B,C). Thus, levetiracetam could be therapeutic in our model.

### Levetiracetam Reduces DNA Damage and Mitigates Synaptic Deficits Caused by Dox

**Figure 9:**
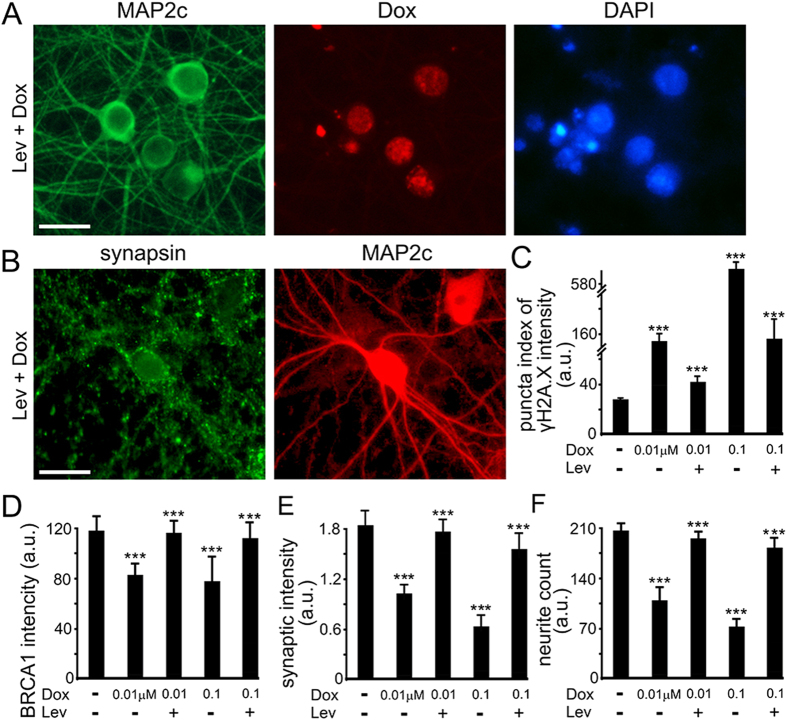
Levetiracetam reduces DNA, synaptic, and neurite damage caused by Dox. (**A**) An example of cortical cultures pre-treated with 5 μM Lev and then Dox (0.01 μM, overnight), fixed and stained with antibodies against MAP2c. Scale bar is 20 μm. (**B**) Cortical neurons at 28–32 DIV were pre-treated with 5 μM Lev, and then a vehicle or with Dox (0.01 μM for 3 days, or 0.1 μM, overnight) was added. Neurons were fixed, and stained for MAP2c, synapsin, and with the Hoechst dye, and imaged. Note that red MAP2c staining also includes Dox’s red fluorescence. (**C**) Synaptically developed primary cortical cultures at 28−32 DIV were pre-treated with 5 mM Lev, and then a vehicle or with Dox (0.01 μM for 3 days, or 0.1 μM, overnight) was added. Neurons were then fixed, and stained with antibodies against γH2A.X and MAP2c, and with the Hoechst dye. Blue staining was used by the algorithm to identify and analyze γH2A.X. (**D**) Quantification of fluorescent images of cortical cultures pre-treated with 5 mM Lev and then a vehicle or with Dox (0.01 μM for 3 days, or 0.1 μM, overnight). Neurons were fixed, and stained with antibodies against BRCA1 and MAP2c, and with the Hoechst dye. Blue staining was used by the algorithm to identify and analyze BRCA1. ***p<0.001 (Dunnett’s test). (**E**) Quantification of fluorescent images of cortical cultures pre-treated with 5 mM Lev and then a vehicle or with Dox (0.01 μM for 3 days, or 0.1 μM, overnight). Neurons were fixed, and stained with antibodies against synapsin and MAP2c, and with the Hoechst dye. MAP2c staining was used by the algorithm to identify and analyze synapses. ***p<0.001 (Dunnett’s test). (**F**) Quantification of fluorescent images of cortical cultures pre-treated with 5 mM Lev and then a vehicle or with Dox (0.01 μM for 3 days, or 0.1 μM, overnight). Neurons were fixed, and stained with antibodies against MAP2c and with the Hoechst dye. MAP2c staining was used by the algorithm to identify and analyze neurites. ***p<0.001 (Dunnett’s test). Several thousand neurons were analyzed. Results were pooled from at least three independent experiments.

To determine if levetiracetam is neuroprotective in Dox-treated neuronal cultures, we pre-conditioned neurons with levetiracetam and then added Dox. Neurons were then fixed and analyzed for γH2A.X, synapsin, and MAP2c. Levetiracetam reduced the formation of DNA DSBs and alleviated synaptic and neurite loss in neurons exposed to Dox (Fig. 9A–D). Thus, in agreement with the beneficial effects of levetiracetam on neurons in models of neurodegeneration and neuronal injury, the deleterious effects of Dox can be reduced by levetiracetam, at least in culture. Future studies in rodents will determine if levetiracetam indeed mitigates cognitive impairments induced by the treatment with Dox. While levetiracetam has not been observed to accelerate brain tumor growth in humans, it will be important to establish that it does not have an adverse effect on either the anti-neoplastic effects of Dox or accelerate non-central nervous system tumor growth.

## Discussion

In this study, we showed that the commonly used chemotherapy drug Dox promotes the formation of DNA DSBs, downregulates the DNA repairing protein BRCA1, and reduces the density of synapses and neurites in cultured primary neurons. We also showed that reducing neuronal activity mitigates Dox-mediated neuronal damage. Dox accumulates in the neuronal nuclei, perhaps damaging the structure of chromatin directly. Remarkably, levetiracetam, an anticonvulsant drug used to treat epilepsy^50^, reduces Dox-mediated formation of DSBs and ameliorates synaptic and neurite abnormalities in primary cultured neurons. A similar result was observed in a model of AD, in which synaptic deficits, DSB, and downregulated BRCA1 were caused by amyloid-beta^15^,^19^. Since co-treatment of cancer patients with anti-neoplastic drugs and neuroprotectants has been proposed as a strategy for preventing and/or alleviating cognitive dysfunction associated with chemotherapy, our search for safe and effective protective drugs in neurons has wide therapeutic implications. Our future studies in mice and rats will determine if levetiracetam alleviates cognitive disturbances induced by Dox.

Dox has restricted access to the brain. However, it appears to cross the blood-brain barrier at levels sufficient to cause damage^13^,^14^,^51^,^52^,^53^,^54^,^55^. In our neuronal model, Dox is toxic, and levetiracetam only partially mitigated damage induced by Dox. As we use levetiracetam as a control to screen for more compounds that may normalize neuronal homeostasis in Dox-treated neurons, we will likely encounter compounds that more effectively reduce Dox-mediated DNA damage and neurotoxicity.

Our data suggest that levetiracetam may be effective in normalizing homeostasis in neurons exposed to many anti-neoplastic agents. For example, abnormal spine density has been observed in neurons treated with cisplatin, a cytotoxic drug used to treat several cancers^56^. Methotrexate, another anti-cancer medication, also affects synapses^57^. Therefore, our results have widespread ramifications for neuroscientists and oncologists who seek to develop neuroprotectants to prevent and treat chemotherapy-induced cognitive impairments in cancer patients and survivors. Levetiracetam appears to be safe in mouse models of AD and in humans^15^,^20^,^58^. Levetiracetam is frequently used as an anti-seizure therapy in patients with brain tumors with few reported adverse side effects other than fatigue. Therefore, levetiracetam may serve as a starter drug for testing therapeutics for chemotherapy-induced cognitive impairments.

## Materials and Methods

### Chemicals

(7S, 9S)-7-[(2R, 4S, 5S, 6S)-4-Amino-5-hydroxy-6-methyloxan-2-yl]oxy-6, 9, 11-trihydroxy-9-(2-hydroxyacetyl)-4-methoxy-8, 10-dihydro-7H-tetracene-5, 12-dione (doxorubicin, Dox), levetiracetam and etoposide were from Selleckchem (Houston, TX). Hoechst dye was from Santa Cruz Biotechnology (#sc-394039). Antibodies against γH2A.X (mouse monoclonal antibody) were from EMD Millipore (clone JBW301; 1:50). Antibodies against MAP2c (#sc-20172; 1:100) were from Santa Cruz Biotechnology. Antibodies against BRCA1 (#ab191042; 1:50), 53BP1 (#ab172580; 1:50), and TBR1 (#ab31940; 1:50) were from Abcam. Tetrodotoxin (TTX) was from Abcam (Cambridge, MA). 2, 3-Dioxo-6-nitro-1, 2, 3, 4-tetrahydrobenzo[f]quinoxaline-7-sulfonamide (NBQX) and D-2-amino-5-phosphonopentanoic acid (APV) were from Cayman Chemical (Ann Arbor, MI). Antibodies against synapsin were a gift from Dr. Roger Janz (University of Texas, Houston) and also were from Synaptic Systems (Goettingen, Germany). Antibodies against Arc were a gift from Dr. M. Neal Waxham (University of Texas, Houston). Anti-rabbit Alexa Fluor 488-labeled, anti-mouse Alexa Fluor 546-labeled, anti-rabbit Alexa 546-labeled, and anti-mouse Alexa 488-labeled secondary antibodies were from Life Technologies (Carlsbad, CA). pDRIVE-hSynapsin-mApple was cloned from the pmApple-C1 construct (a gift from Dr. Kurt Thorn, University of California, San Francisco)^59^ and the pDRIVE plasmid bearing human SYN1 promoter (InvivoGen, San Diego, CA).

### Cell Cultures

Cortices from rat embryos (E17–18) were dissected, dissociated, and plated on 96-well tissue-culture plates (0.5–1×10^5^/well) or on 24-well tissue-culture plates (0.2–0.7×10^6^/well) coated with poly-D lysine as described^24^,^26^,^60^,^61^,^62^. Neurons were grown in modified neuronal growth medium made from Neurobasal Medium (Life Technologies), B-27 supplement (Life Technologies) or SM1 supplement (STEMCELL), GlutaMAX (Life Technologies), and penicillin-streptomycin (Life Technologies) for 1 month. Although no serum is present in our media, glial cells still proliferate and become confluent after about 2–3 weeks in culture. We do not add an inhibitor of glial proliferation to the media. Some cultures were transfected with the pDRIVE-hSynapsin-mApple (Lipofectamine 2000, Invitrogen) at 4–5 DIV. Although some transfection protocols suggest that it is not necessary to remove the DNA:Lipofectamine 2000 complexes, we always remove complexes from neuronal cultures after 0.5–1 h. Longer incubations with Lipofectamine 2000 result in significant toxicity. Low transfection efficiency is not a problem for our analyses. We routinely obtain hundreds of transfected neurons per 96-well plate and even more for 24-well plate.

### Treatments

Neurons were treated with 0.01–1 μM Dox and 5 μM etoposide for various intervals (see figure legends). Some neurons were pre-treated with 5 μM levetiracetam for 24 hours and then with Dox (see figure legends). Some neurons were co-treated with 0.05 μM Dox and TTX (1 μM), or NBQX (50 μM) or APV (100 μM) overnight. Some cultures were treated with 1–5 μM levetiracetam. Due to a possible variation between neuronal preparations, all conditions including controls were compared within an experiment.

### Immunocytochemistry

For visualizing DNA DSBs, cultured neurons were fixed with ice-cold methanol for 10 min, and then 4% paraformaldehyde for 5 min at room temperature. Some cultures were also fixed with warm 4% paraformaldehyde for 15 min. Fixed cultures were blocked for 1 hour in PBS containing 10% serum from the host species of a secondary antibody. Neurons were incubated with antibodies against γH2A.X (1:50) or 53BP1 (1:50) and MAP2c (1:100) diluted in blocking buffer at 4 °C. Cells were then washed with blocking buffer, and incubated with a secondary antibody in blocking buffer for 1 hour at room temperature. Nuclei were stained with Hoechst dye in PBS.

For visualizing synapses, 4-week-old neurons were fixed with warm 4% paraformaldehyde for 15 min and processed as described above. For visualizing BRCA1, 4-week-old neurons were fixed with 4% paraformaldehyde for 15 min and processed as described above.

We decided to use the immunocytochemistry approach to measure protein expression, rather than western blotting, because 1) significantly less reagents (e.g., antibodies) as well as neurons are required to do an experiment in a 96-well or 24-well plate; 2) the imaging approach is significantly more sensitive, reproducible, and faster; 3) this method also allows us to analyze the subcellular localization of a protein of interest; and 4) neurons may react to the chemotherapy drugs differently than glial cells, which are present in our cultures, and neuronal effects can be masked by glial effects.

Doxorubicin was visualized with the RFP filter. Note that longer incubations of fixed cells in a blocking or washing buffers reduce the red doxorubicin staining. We also found no evidence that doxorubicin affects MAP2c staining and following staining with the red ALEXA 546 dye.

### Fluorescence Microscopy and Image Analysis

Imaging was performed with the EVOS microscopy system (Life Technologies). Briefly, fixed neurons were imaged in automated fashion. The plate was placed on the EVOS microscope stage, which automatically positions the 20× objective to the center of the first well and collects fluorescence images with the RFP filter (mApple; MAP2c), the GFP filter (γH2A.X; 53BP1; synapsin; BRCA1; TBR1), and the DAPI filter (Hoechst), thereafter moving the stage to each adjacent field in the well. These steps are repeated until all required wells are imaged. Images were analyzed with the EVOS software, ImageJ, and custom MatLab software^25^.

To measure the synaptic and neurite changes, we used the Neuronal Profiling V4 BioApplication software (Thermo Scientific). Hoechst staining was used by the algorithm to identify nuclei. Synapsin staining was used by the algorithm to identify and analyze synapses. MAP2c staining was used by the algorithm to identify and analyze neurites. The neurite count was determined with the NeuriteTotalCount per field algorithm and represented in arbitrary units.

Puncta indexes were analyzed as described^24^. Briefly, γH2A.X fluorescence intensity was reflected by the puncta index, which is the standard deviation of the intensities measured among pixels within the nuclear region (detected with Hoechst dye). Diffuse and low fluorescence intensity corresponds to a low puncta index, and punctate and high fluorescence corresponds to a high puncta index.

Several thousand neurons were analysed per condition. Experiments were repeated at least three times.

### Longitudinal Fluorescent Microscopy and Survival Analysis

To measure neurotoxicity quantitatively, several large cohorts of neurons were followed over time. Neurons were transfected with a red morphology and viability marker pDRIVE-hSynapsin-mApple, treated with vehicle (DMSO) or with a drug 24 hours after transfection, and imaged immediately and then every 24 hours for several days. The plate was placed on the EVOS microscope stage, which automatically positions the 20× objective to the center of the first well and collects fluorescence images with the RFP filter (mApple), thereafter moving the stage to each adjacent field in the well. These steps were repeated until all required wells were imaged. For tracking the same neurons over time, an image of the fiduciary field with neurons on the plate was collected at the first time-point and used as a reference image. Each time the same plate was imaged thereafter, the fiduciary image was aligned with the reference image. Neurons that died during the imaging interval were assigned a survival time (the period between transfection and their disappearance from an image). Cells that survived the entire experiment are weighted differently to account for an indeterminate survival time. These event times were used to obtain the exponential cumulative survival graphs and analyzed for statistical significance by Log-Rank test. A hazard function, which describes the instantaneous risk that a neuron from the cohort will reach the endpoint of interest, was obtained from the survival times. R and JMP (SAS Institute, Cary, North Carolina) were used for statistical analyses used as described^26^,^61^,^62^. Curves were generated in JMP. The cumulative nature of the analysis makes it much more sensitive (100–1000×) than approaches that which depend on averaged responses measured at particular time points^63^. Experiments were repeated at least three times.

### Ethics Statement

Rats were maintained in accordance with guidelines and regulations of the University of Texas, Houston (the protocol number #AWC-13-122). All experimental protocols were approved by the University of Texas, Houston. The methods were carried out in accordance with the approved guidelines.

## Additional Information

**How to cite this article**: Manchon, J. F. M. *et al.* Levetiracetam mitigates doxorubicin-induced DNA and synaptic damage in neurons. *Sci. Rep.* **6**, 25705; doi: 10.1038/srep25705 (2016).
